# Food Liking-Based Diet Quality Indexes (DQI) Generated by Conceptual and Machine Learning Explained Variability in Cardiometabolic Risk Factors in Young Adults

**DOI:** 10.3390/nu12040882

**Published:** 2020-03-25

**Authors:** Ran Xu, Bruce E. Blanchard, Jeanne M. McCaffrey, Stephen Woolley, Lauren M. L. Corso, Valerie B. Duffy

**Affiliations:** 1 Department of Allied Health Sciences, University of Connecticut, 358 Mansfield Rd, Storrs, CT 06269, USA; ran.2.xu@uconn.edu (R.X.); bruce.blanchard@uconn.edu (B.E.B.); jeanne.mccaffery@uconn.edu (J.M.M.); lauren.lamberti@uconn.edu (L.M.L.C.); 2 Institute of Living, Hartford Hospital, 200 Retreat Ave, Hartford, CT 06106, USA; stevewo2003@yahoo.com

**Keywords:** diet, diet quality, cardiometabolic health, metabolic syndrome, young adult, principal component analysis, food preference, ridge regression analysis, vegetables, sweets

## Abstract

The overall pattern of a diet (diet quality) is recognized as more important to health and chronic disease risk than single foods or food groups. Indexes of diet quality can be derived theoretically from evidence-based recommendations, empirically from existing datasets, or a combination of the two. We used these methods to derive diet quality indexes (DQI), generated from a novel dietary assessment, and to evaluate relationships with cardiometabolic risk factors in young adults with (*n* = 106) or without (*n* = 106) diagnosed depression (62% female, mean age = 21). Participants completed a liking survey (proxy for usual dietary consumption). Principle component analysis of plasma (insulin, glucose, lipids) and adiposity (BMI, Waist-to-Hip ratio) measures formed a continuous cardiometabolic risk factor score (CRFS). DQIs were created: theoretically (food/beverages grouped, weighted conceptually), empirically (grouping by factor analysis, weights empirically-derived by ridge regression analysis of CRFS), and hybrid (food/beverages conceptually-grouped, weights empirically-derived). The out-of-sample CRFS predictability for the DQI was assessed by two-fold and five-fold cross validations. While moderate consistencies between theoretically- and empirically-generated weights existed, the hybrid outperformed theoretical and empirical DQIs in cross validations (five-fold showed DQI explained 2.6% theoretical, 2.7% empirical, and 6.5% hybrid of CRFS variance). These pilot data support a liking survey that can generate reliable/valid DQIs that are significantly associated with cardiometabolic risk factors, especially theoretically- plus empirically-derived DQI.

## 1. Introduction

Young adults can show risks for type 2 diabetes and cardiovascular disease with a clustering of biological risks including prediabetes and metabolic syndrome. From the National Health and Nutrition Examination Survey (NHANES 2005–2006 through 2015–2016), the rate of pre-diabetes was 16% in young adults, and pre-diabetes rates were associated with elevated central adiposity and systolic blood pressure, less favorable levels of non-high-density lipoprotein cholesterol, and lower insulin sensitivity [1]. Furthermore, the rates of metabolic syndrome in young adults were 23.2% from NHANES 2007–2012 [2]. Interestingly, previous evidence shows that the relative importance of the components that comprise the metabolic syndrome differs from young adults to later adulthood [3,4]. The components of the metabolic syndrome tend to correlate, and cardiometabolic risk scores have been suggested as a more sensitive and specific approach to assess cardiometabolic health and chronic disease risk relationships [4,5,6,7].

Greater abdominal or trunk adiposity in young adults results in greater risk of cardiometabolic risk in later adulthood [8,9]. Healthy behaviors, such as physical activity [10] and a healthy overall diet, can decrease the risk of cardiometabolic disorders. The healthiness of the diet or diet quality may provide a better predictor of disease risk [11] than specific nutrients [12] or restricted food patterns [13]. However, the index of diet quality must be sensitive enough to associate with chronic disease risk factors in young adults [14]. There is a need for examining the scoring and construction of diet quality indexes to improve the understanding of diet-cardiovascular disease risk [15]. The present study will focus on new methodologies to assess habitual food and beverage consumption and calculation of a diet quality index that may enhance the ability to see these relationships in young adults.

Measures of diet quality involve scoring reported consumption of foods or food groups into a single index, such as the Healthy Eating Index (HEI) [16] or the Diet Inflammatory Index (DII) [17], and adjusting the foods, food groups, or index for total energy consumed to capture quality instead of quantity consumed. The ability of the diet quality index to advance evidence on nutrition and health is dependent on capturing habitual dietary consumption (including total energy intake) with a scoring algorithm that is evidence based. The question of interest is if we can simplify the measure of dietary consumption and scoring and obtain an index that is predictive of diet-related measures of health and chronic disease in young adults.

Usually, diet quality indexes are derived from self-reported measures such as 24-h recalls, food records/diaries, or food frequency questionnaires (FFQ). It is difficult to accurately measure usual intake, including total energy intake [18]. The measures may only capture intake on the short-term (e.g., 24-h recall), missing day-to-day variation in food and energy intakes. Dietary histories or FFQ increase the likelihood of capturing usual intake, but may suffer from over/under reporting bias, difficulty of remembering the type and frequency of foods consumed, challenges of converting what is consumed into frequency categories, and participant and researcher cost/time [19]. Liking surveys show promise as a tool to assess dietary intake [20,21,22,23] and for examining related health outcomes [24] based on the assumption that, over time, we tend to eat what we like and avoid what we do not. In some scientific literature, the terms food preference or liking are used interchangeably with food consumption (e.g., [25,26]). Food liking surveys are easy for participants to complete, rapid to process, and can correlate with self-reported intake [21,27,28,29,30,31] or intake assessed with biomarkers [21,32,33]. Marginal associations between liking and consumption or body weight [34] may indicate dietary restraint [21,30,35].

With the dietary data available, the next step is how to combine the foods or food groups into a diet quality index that represents the totality of the foods and beverages habitually consumed [36]. Theoretically driven or *a priori* assumptions are based on an evidence analyses of healthy eating research [37] such as the HEI from the Dietary Guidelines for Healthy Americans [16], the diet quality index for adolescents based on nutrient recommendations of the Belgian Health Council [38] or the DII based on extensive review of diet components associated with inflammatory biomarkers [17]. For example, the HEI is based on weighting nine adequacy components and four moderation components in five- and 10-point increments to come up with a 0 to 100 score. We created a diet quality score from the food liking survey with conceptual weights based on Dietary Guidelines for preschoolers [39] and adults [30,33], showing significant associations between this index, dietary biomarkers, and body weight. As diet quality indexes should be independent of energy consumed, we have shown that the liking-based diet quality index is independent of energy consumed in studies with preschoolers [39] and adults [30]. Data driven or *a posteriori* methods describe statistically-generated dietary patterns through interrelationships between and identification of underlying components (e.g., principal component analysis) or by identifying unique clustering of dietary patterns (e.g., cluster analysis) [37] and evaluating the ability to explain health-related outcomes such as risk of overweight or obesity [40,41]. Finally, hybrid methods combine the theoretical with data driven approaches to define measures that maximize the explanation of predictor variables including biomarkers of disease [42], obesity risk [43], and functional status [44]. It is unknown if empirically and hybrid driven data patterns derived from food liking surveys would associate with disease risk predictor variables.

Thus, the objectives of the present study were two-fold. The first objective was to compare methods (i.e., theoretical, empirical, and a hybrid of theoretical and empirical) of constructing diet quality indexes (DQI) from a food liking survey to predict cardiometabolic risks. The second objective involved the DQI that was most predictive of the cardiometabolic risk and transformation to a 0–100 scale. This transformation allowed the evaluation of the relative importance of each food group for cardiometabolic risk in comparison with the Healthy Eating Index and current dietary recommendations.

## 2. Materials and Methods

### 2.1. Participants

This observational study enrolled 106 English-speaking patients (non-diabetic) with diagnosed depression between 18–25 years old, recruited at psychiatric institutions that are members of the Hartford Healthcare Behavioral Health Network (Hartford, CT), and 106 non-depressed controls from the University of Connecticut (Storrs, CT). The primary goal of the participant recruitment was to assess risks of metabolic syndrome in young adults with depression who were treated with second-generation antipsychotics. The present study leveraged these data to examine the ability of the diet quality indexes to predict cardiometabolic risks within a study sample of young adults who had variability in these risks.

The study was conducted in accordance with the Declaration of Helsinki and the study procedures were Internal Review Board (IRB) approved at Hartford Hospital (Hartford, CT) and the University of Connecticut (Storrs, CT). The patients were informed of the research after eligibility was affirmed via reviewing the automated data and charts for diagnosis of a major depressive disorder, currently receiving in-patient or out-patient care, treatment with second-generation antipsychotics, not pregnant, and not diagnosed with diabetes mellitus. A member of the research team read a detailed description of the study. Controls who were non-diabetic and without a self-reported history of mental illness and/or treatment were recruited for voluntary participation by IRB-approved posters and electronic announcements at the University. If eligible and voluntarily agreeing, all participants read and signed approved informed consent and HIPAA forms, and were paid for participating. All participants visited a treatment or laboratory-based testing facility once for completion of health and wellness (e.g., sleep, stress, eating, exercise) interviews and physical measurements, and then a second time for a fasting venipuncture.

### 2.2. Study Procedure and Measures

The study procedures and measures were completed in two sessions. In the first session and after informed consent, participants answered questions and completed surveys related to demographics, personal and family health, stress and stress-related problems, quality of sleep, lifestyle (e.g., diet/nutrition, physical activity, alcohol and tobacco use), and depression. Following the interviews, anthropometric measurements were obtained via a non-invasive physical examination. The second session included the fasting blood collection.

Liking Survey: Each participant completed a paper/pencil version of our validated liking survey [32]. The survey was labeled “Your Likes and Dislikes,” with instructions for the participant to report their likes and dislikes for foods, physical activity, and common experiences anywhere along a horizontal line scale labeled above first with three adjectives (far left with “strongest disliking of any kind”, in the middle with “neither like nor dislike”, and to the far right, “strongest liking of any kind”) and five faces (i.e., facial hedonic scale). The participant was shown an example of a rating for “walking out of a dark movie on a bright sunny day” that fell in the middle of the disliking part of the scale and then asked to report their liking for “hearing your favorite piece of music” and then “glare of headlights at night.” Next, they were asked to report and rate the level of liking/disliking for their favorite and least favorite food and then favorite and least favorite physical activity. Finally, they were asked to report the level of liking for 92 items for foods, physical activities, and common pleasant and unpleasant sensations shown as a picture and a word. Thus, the directions reinforced that the scale could be used to report liking broader than foods. The items were scored with a ±100-point ruler, where 0 was neither like nor dislike, ±75 to 100 was strongest liking/disliking of any kind with the biggest smile or crying face, ±30 to 60 was the intermediate smile or dislike face. The food and beverage items were selected by nutrition researchers in a multi-country collaborative project [32] to have three items each for conceptual food groups to represent the whole diet with addition of items that captured specific sensory qualities (e.g., sour).

Anthropometric Measurements: During a non-invasive physical examination, anthropometric measurements were performed. Obesity/adiposity measurements were obtained in multiple ways. Waist and hip circumference measurements (in cm) were performed in triplicate at three different anatomical locations: iliac crest, at the narrowest waist, and around the maximum protrusion of the buttocks. From these measurements, waist to hip ratios were calculated. Circumference measurements were supplemented by height and weight for the calculation of body mass index (BMI). All measurements were performed in triplicate and averaged to ensure reliability of results.

Cardiometabolic Biomarkers: Fasting blood samples were obtained via venipuncture to provide measures of insulin, glucose, and lipids (i.e., triglycerides, total cholesterol, HDL, LDL). All biomarker analysis was performed by certified medical laboratory scientists on automated chemistry analyzers at a clinical hospital laboratory (Hartford Hospital; Hartford, CT).

Cardiometabolic Risk Factor Scores (CFRS): Although cardiovascular disease risk factors tend to correlate in the general population [45], there also are reports of different patterns of risks in young adults [4], including those with special conditions [46], and the advantage of creating a continuous risk score with statistical grouping to associate with dietary behaviors [47] instead of a dichotomous yes/no with metabolic syndrome as in clinical applications [48].

To construct a continuous CFRS, we first performed pairwise correlation analysis and exploratory factor analysis with varimax rotation on plasma (insulin, glucose, HDL cholesterol, triglycerides), adiposity (BMI, Waist-to-Hip ratio), and blood pressure (systolic and diastolic blood pressure) measures of each participant, and retained measures that loaded moderately or highly on the same principle factor. We then performed principle component analysis (PCA) to generate a continuous cardiometabolic risk factor score based on the retained measures. Each plasma, adiposity, and blood pressure measure was first transformed to approach normal distribution and then standardized before the analysis. The total percentage of variance explained by PCA was reported.

### 2.3. Diet Quality Index Methods

Theoretically-constructed Diet Quality Index (DQI): The food items were averaged into conceptual groups as shown in Table 1, with most having acceptable internal reliability. Ten of the nutritional groups were considered for the theoretical DQI. The items of the groups were averaged and then weighted based on healthiness following Dietary Guidelines 2015 [49]: sweet/sugary beverage (−3), fruit (+2), vegetable (+3), refined carbohydrate (−1), healthy fat/seafood (+3), spicy/flavorful (+2), low-fat protein (+3), high-fat protein (−3), salty (−2), and complex carbohydrate (+2). Higher scores on the indices indicated healthier behaviors. Thus, we had a balance of weights for healthy versus unhealthy foods/beverages, as seen with other diet quality indexes [15].

Empirically-constructed Diet Quality Index (DQI): All of the individual items for food and beverages from the food liking survey were considered to construct the empirical DQI. We first performed factor analysis with varimax rotation on all of the items for food and beverages, and obtained the top 10 factors that explained the most variance. Then, we performed statistical analysis and empirically generated the weight of each factor that best predicts the CFRS. The ridge regression model was chosen to maximize the interpretability of the model while avoiding overfitting on the training data [50,51] and preserving out-of-sample predictive power. Ridge regression is essentially a linear regression model with a penalty/regularization term. The linear form ensures the interpretability of the weight for each predictor and the regularization term penalizes large coefficients to avoid overfitting on the training data. Specifically, we performed ridge regression with CFRS as the outcome and the 10 factors as the primary predictors of interest, while also controlling for male/female, patient status, and pleasant and unpleasant experiences of each subject. The pleasant and unpleasant experiences served as non-food standards for liking ratings [52,53]. Regularization parameter λ was chosen via cross validation on the training data to minimize the mean-squared prediction error. Empirical DQI was constructed as the weighted sum of the 10 factors, with weights equal to the regression coefficient of each factor in final the ridge regression model. Finally, empirical DQI was reversely coded such that higher DQI will associate with lower cardiometabolic risks.

Hybrid Diet Quality Index (DQI): All of the conceptual food and beverage nutritional groups (vegetable, fruit, salty, high-fat protein, alcohol, complex carbohydrate, sweet/sugary beverage, healthy fat/seafood, low-fat dairy, refined carbohydrate, saturated fat, spicy/flavorful) were considered as the basis to construct the hybrid DQI. Similar to the empirical DQI, we empirically generated weight of each nutritional group that best predicts the CFRS. Ridge regression model was chosen to maximize interpretability while avoiding overfitting on the training data and preserving predictive out-of-sample power. Specifically, we first standardized all the nutritional groups. Then we performed ridge regression with CFRS as the outcome and the updated nutritional groups as the primary predictors of interest, while also controlling for male/female, patient status, and pleasant and unpleasant experiences of each subject. Similarly, regularization parameter λ was chosen via cross validation on the training data to minimize the mean-squared prediction error. Hybrid DQI was constructed as the weighted sum of the nutritional groups, with weights equal to the regression coefficient of each nutritional group in the final ridge regression model. Finally, hybrid DQI was reversely coded such that higher DQI will associate with lower cardiometabolic risks.

### 2.4. Statistical Analyses

Predicting CFRS: Comparisons between the DQIs: To evaluate the criterion validity of various DQIs, we assessed each DQI’s out of sample predictive power on CFRS by performing two-fold and five-fold cross validations respectively. In a k-fold cross validation, the original sample is randomly partitioned into k equal sized subsamples, each DQI was generated based on procedures described above using k-1 subsamples, and the predictive power of each DQI on CFRS was assessed based on the subsample that was not used. Specifically, for each DQI we performed two multivariate regressions on the subsample, both using CFRS as the outcome and male/female, patient status, and pleasant and unpleasant experiences as predictors, but one with the DQI as an additional predictor and the other without. The difference in the total variance of CFRS explained (R-square) between the two models thus represents the additional variance explained by the DQI, which can be interpreted as the predictive power of that DQI. In a k-fold cross validation, we performed this procedure k times and obtained the average additional variance of CFRS explained by each DQI.

Scale standardization and relative importance of the nutritional group: To allow direct comparison between the theoretical and hybrid DQI and interpretation of the clinical importance of each nutritional group, we transformed the hybrid DQI to a 0–100 scale (similar to the HEI). Specifically, we first re-coded all the nutritional groups to a 0–100 scale, and the nutritional groups that have positive relationships with CFRS are reversely coded. We then transformed the standardized weights (i.e., standardized regression coefficient from the ridge regression) of the nutritional groups such that the transformed weights sum up to 1. The new hybrid DQI is then the weighted sum of the transformed liking scores of the nutritional groups (with the transformed weights) and ranges from 0–100. The transformed weights of the hybrid DQI were compared across nutritional groups to assess the relative importance of each nutritional group, and were compared with weights of each nutritional group in theoretical DQI (weighted based on healthiness following Dietary Guidelines 2015) to assess the accordance of hybrid DQI with these guidelines. The *p*-values of the key statistics were reported and all data analyses were conducted in STATA 16.0.

## 3. Results

### 3.1. Descriptive Findings—Cardiometabolic Risk Factors and Food Liking

**Figure 1:**
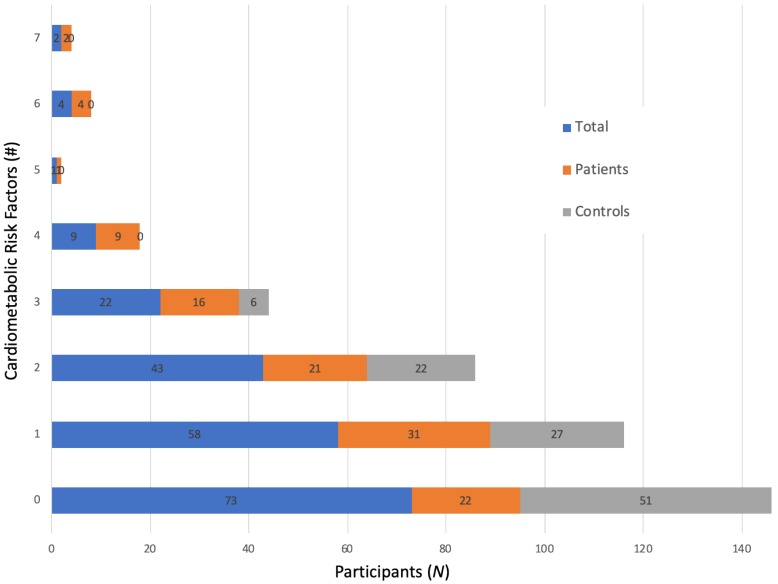

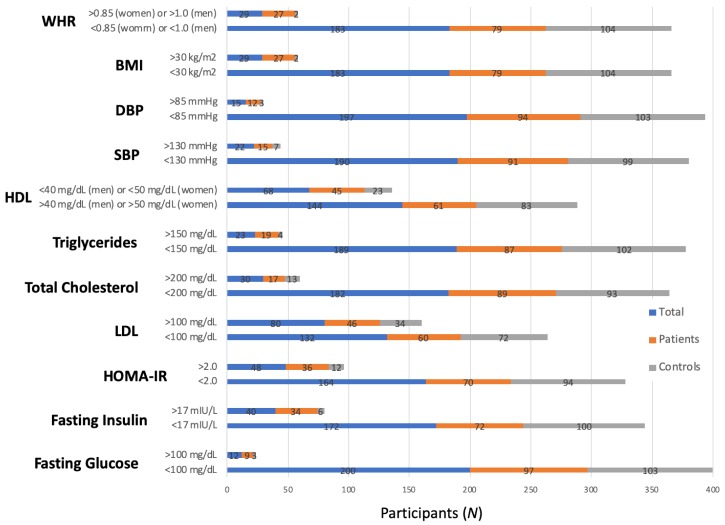
Variability in the Cardiometabolic Factors for the Total Sample, Patients with Major Depressive Disorder, and Student Control (*n* = 212). Data are presented as the number of cardiometabolic abnormalities present for the total, patient, and control samples (top) and normal versus abnormal values for each cardiometabolic factor for the total, patient, and control samples (bottom). Factors are treated as independent, continuous determinants of metabolic health. Normal values for cardiometabolic factors were considered the following; BMI <30 kg/m^2^. SBP/DBP <130/<85 mmHg; fasting glucose <100 mg/dL; fasting insulin <17 mIU/L; total cholesterol <200 mg/dL; LDL-C <100 mg/dL; Triglycerides <150 mg/dL, HDL > 40 mg/dL (men) or >50 mg/dL (women); WHR <0.85 (women) or <1.0 (men); HOMA-IR (HOMA2) < 2.0. *Abbr.* BMI= body mass index. DBP = diastolic blood pressure. HDL-C= high density lipoprotein cholesterol. HOMA-IR= homeostatic assessment model of insulin resistance. LDL-C= low density lipoprotein cholesterol. SBP = systolic blood pressure. WHR = waist-to-hip ratio.

On average, the sample was comprised of young (mean ± SD; 20.8 ± 1.9 years), predominantly white/Caucasian, male (*n* = 80, 37.7%) and female (*n* = 132, 62.3%), overweight (25.3 ± 6.9 kg/m^2^) young adults (Table 2). The sample showed variability in the cardiometabolic factors. Tests for skewness and kurtosis showed that the cardiometabolic factors significantly deviated from a normal distribution. Sixty-five percent (*n* = 139) of participants had the presence of at least one abnormal cardiometabolic factor (Figure 1). Participants with major depressive disorder had more cardiometabolic abnormalities than did healthy student controls (*p* < 0.001, Figure 1). Only 10 (4.7%) participants met the standard NCEP ATPIII criteria for metabolic syndrome.

Pairwise correlations showed plasma (insulin, glucose, HDL cholesterol, triglycerides) and adiposity (BMI and Waist-to-Hip ratio) measures were moderately to strongly correlated with each other (average correlation = 0.32, each measure had a correlation >0.3 with at least one other measure), while they were only weakly correlated with the blood pressure (systolic and diastolic) measures (average correlation = 0.12). Exploratory factor analysis further confirmed the intuition and showed that plasma and adiposity measures loaded moderately to highly on the first factor, while blood pressure loaded highly on the second factor. Thus, plasma and adiposity measures were retained and formed the basis of the combined cardiometabolic risk factor score. Insulin, glucose, HDL cholesterol, triglycerides, and BMI were transformed to approach a normal distribution. The first component from the principle component analysis (PCA) explained 44.2% of the total variance in these variables, which was then used as the CFRS.

**Figure 2:**
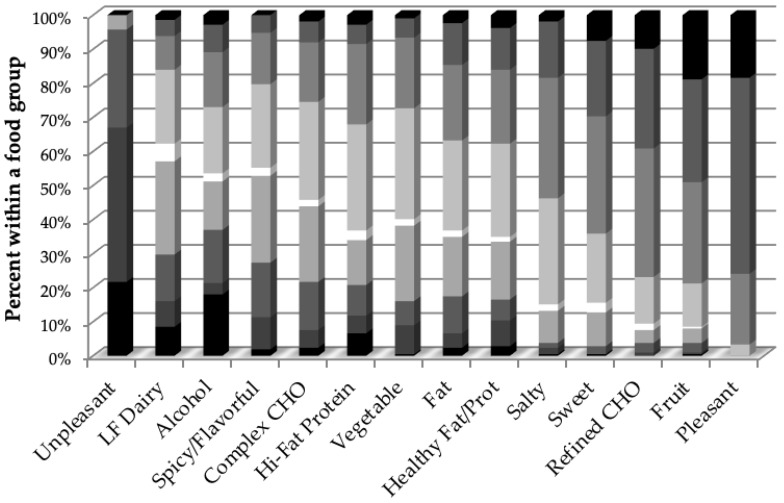
Percent of young adults within a food group from Table 1 who reported like (above the white neutral rating) and dislike (below the white neutral rating), partitioned into 25th percentiles, with the darker the shading, the stronger the liking or disliking. The food groups are ordered from least to most liked bracketed by unpleasant and pleasant non-food sensations. (CHO = carbohydrate, LF = low-fat)

From the individual foods/beverages, those averaging below neutral (dislike) and at the bottom quartile were tomato juice, low-fat cottage cheese, blackened hot dog, dark beer, horseradish, bologna, black coffee, oatmeal, canned tuna in water, tabasco sauce, mayonnaise, scotch, grapefruit juice, sautéed mushrooms, burn of a spicy meal, and skim milk (listed from most to least disliked). Those averaging liked at the top quartile were cheesecake, cheddar cheese, tortilla chips, chocolate milk, baked chicken, bagel or rolls, French fries, blueberry, vanilla ice cream, pineapple, mango, spaghetti/pasta, cheese pizza, cookies, cakes or pastry, and strawberries (listed from liked to most liked). The most liked groups were pleasant activities (items include hearing your favorite piece of music, smell of freshly cut grass, favorite food, favorite physical activity, seeing a loved one completing a challenging task, cooling off on a hot day, watching TV), fruit, refined carbohydrate, and sweet/sugary beverage (listed from highest); least liked were unpleasant activities (items include being caught in a lie, glare, most disliked physical activity, least favorite food, fear of being bitten by a snake), low-fat dairy, spicy/flavorful food, and complex carbohydrates (listed from disliked to barely liked). The reported liking of the food groups also showed good variability (Figure 2).

Shown in Table 3, many of the nutritional groups were moderately or highly correlated with each other, such as salty and high fat proteins, (r = 0.66, *p* < 0.001), or sweet, saturated fat and refined carbohydrates. To avoid multi-collinearity when constructing the hybrid DQI, for nutritional groups that had a correlation larger than 0.5, we combined them into one group by taking the average. The procedure was performed to combine salty and high-fat proteins, and the same procedure was performed for sweet, saturated fat and refined carbohydrates groups. This resulted in a total of nine updated nutritional groups as the input for the hybrid DQI.

### 3.2. Descriptive Findings—Diet Quality Indexes

**Figure 3:**
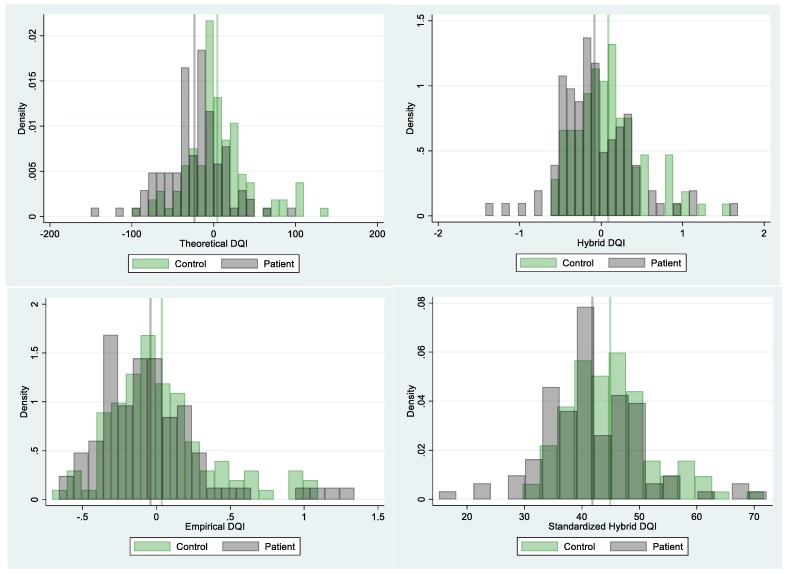
Histograms of the density of young adults with (patients) and without (controls) diagnosed depression by diet quality indexes calculated from food liking/disliking responses and formed by theoretically (based on dietary guidelines), empirically (based on factor analysis), and hybrid (based on conceptual and statistical approaches) approaches and the hybrid approach standardized to a 100-point scale.

Figure 3 shows the distribution of diet quality indexes (DQI) in the young adult patient and controls.

Theoretical DQI (top left panel, Figure 3) showed considerable variability across the sample (Mean = −9.36, SD = 40.65). Kolmogorov-Smirnov tests showed that the overall distribution of theoretical DQI was not significantly different from the normal distribution (*p* = 0.152). The patients (grey) had significantly lower average theoretical DQI than that of the control (green) group (D = 27.93, *p* < 0.001). Principal Component Analysis revealed two dimensions that could be labeled into healthy and less healthy and explained nearly 52% of the variability across all groups. The theoretical DQI approached adequate internal reliability (alpha = 0.673).

Empirical DQI (bottom left panel, Figure 3) also showed considerable variability across the sample (Mean = 0.001, SD = 0.36). However, the overall distribution was somewhat skewed and Kolmogorov-Smirnov tests showed the distribution of empirical DQI was significantly different from the normal distribution (*p* = 0.04). The patients (grey) also had a lower mean than that of the control (green) group, but the difference was not statistically significant under 0.05 significance level (D = 0.077, *p* = 0.15). The top 10 factors from factor analysis with varimax rotation explained 54% of variation among all individual food liking survey items. The resulting factors had moderate consistency with the nutritional groups but often included individual food items from different nutritional groups. For example, the top factor had high loadings on some but not all items from the sweet, refined carbohydrates groups and saturated fat, the second factor had high loadings on some items in the salty and high fat protein group, and the third factor had high loadings on some items in the healthy fat group.

Hybrid DQI (top right panel, Figure 3) also showed considerable variability across the sample (Mean = 0.004, SD = 0.435). Kolmogorov-Smirnov tests showed that the overall distribution of hybrid DQI was not significantly different from the normal distribution (*p* = 0.217). The patients (grey) had a lower mean than that of the control (green) group and the difference was statistically significant (D = 0.17, *p* = 0.005). The relative importance (from high to low) of each nutritional group based on the magnitude of the ridge regression coefficients was: sweet, fat, and refined carbohydrates, vegetable, complexed carbohydrates, spicy/flavorful, alcohol, low-fat dairy, fruit, salty and high-fat protein, and healthy fat. As hybrid DQI was constructed based on standardized nutritional groups and empirical DQI was constructed based on factors from individual food items, the three DQIs had different scales and were not directly comparable in terms of mean and standard deviation. However, correlations between the three DQIs were moderate to strong. Specifically, in our sample, the correlation was 0.7 between hybrid DQI and empirical DQI, 0.55 between theoretical DQI and empirical DQI, and 0.45 between hybrid DQI and theoretical DQI.

### 3.3. Predicting CFRS: Comparisons between the DQIs

Results from two-fold cross validations showed that on average regression model including theoretical DQI, patient status, male/female, and pleasant and unpleasant experiences explained 24.5% of the variance in CFRS in the testing data, and theoretical DQI explained 1.1% variance in CFRS in the testing data, after controlling for patient status, male/female, and pleasant and unpleasant experiences. For empirical DQI, two-fold cross validation results showed that, on average, the regression model including empirical DQI, patient status, male/female, and pleasant and unpleasant experiences explained 25.2% of the variance in CFRS in the testing data, and empirical DQI explained 1.8% variance in CFRS in the testing data after controlling for patient status, male/female, and pleasant and unpleasant experiences. Finally, two-fold cross validation results showed that, on average, the regression model including hybrid DQI, patient status, male/female, and pleasant and unpleasant experiences explained 29.2% of the variance in CFRS in the testing data, and hybrid DQI explained 5.8% variance in CFRS in the testing data after controlling for patient status, male/female, and pleasant and unpleasant experiences.

Five-fold cross validations showed a similar pattern. Specifically, on average, the overall regression model including theoretical DQI, patient status, male/female, and pleasant and unpleasant experiences explained 32.6% variance in CFRS in the testing data, and the theoretical DQI explained 2.6% variance in CFRS in the testing data. The overall regression model including empirical DQI, patient status, male/female, and pleasant and unpleasant experiences on average explained 32.7% variance in CFRS in the testing data, while the empirical DQI explained 2.7% variance in CFRS in the testing data. Finally, on average, the overall regression model including hybrid DQI, patient status, male/female, and pleasant and unpleasant experiences explained 36.4% variance in CFRS in the testing data, while the hybrid DQI explained 6.5% variance in CFRS in the testing data.

Overall, these results suggested that the hybrid DQI had highest predictive power of CFRS and was able to explain 5.8%–6.5% of the variation in CFRS in the testing data, followed by empirical DQI (1.8%–2.7%) and theoretical DQI (1.1%–2.6%).

### 3.4. Standardized Hybrid DQI Components and Scoring Standards

The standardized hybrid DQI score was distributed approximately normal (Mean = 43.33, SD = 8.22, *p*-value for Kolmogorov-Smirnov tests: 0.316), and the mean for the control group (44.93) was significantly higher than the patient group (41.81, *p* = 0.006). The relative importance of each nutritional group is presented in Table 4. For example, if a person scores 100 on vegetable group in the liking survey, he/she will have a maximum score of 18. In contrast if the person scores −100 on vegetable group in the liking survey, he/she will have a minimum score of 0. If a person has a maximum score on every nutritional group in this table, he/she will have a total score of 100, which represent the lowest cardiometabolic risk.

The relative importance of each nutritional group in the standardized hybrid DQI was ranked as (high to low): sweet, fat, and refined carbohydrates, vegetable, complex carbohydrates, spicy/flavorful, alcohol, low-fat dairy, fruit, salty and high fat protein, and healthy fat. The relative importance in the standardized hybrid DQI had moderate consistency with the Dietary Guidelines and the weight of the theoretical DQI. For example, both scoring standards gave large positive weights to vegetables, and relatively large negative weights to sweet, fat, and refined carbohydrates. However, there were also components that are qualitatively different. The salty and high fat protein group was much less important in hybrid DQI than in Dietary Guidelines once sweet, fat, and refined carbohydrates were included, and the complexed carbohydrates group had a non-neglectable negative weight in hybrid DQI compared with the Dietary Guidelines. This shows that the liking for the complex carbohydrates group may actually positively associate with cardiometabolic risk.

## 4. Discussion

Nutrition and public health experts and guidance recognize that the healthiness of the overall diet contributes to the prevention of chronic disease. The present observational study used a simple liking survey to create indexes of diet healthiness or diet quality and examined their ability to explain variability in cardiometabolic risk factors in young adults. The index that was a hybrid of theoretically-driven (i.e., based on scientific evidence and current guidelines) as well as data-driven explained the most variability in a cardiometabolic risk factor score (CRFS; plasma lipids, glucose, adiposity). The hybrid index included conceptually-based food and beverage groups that were weighted based on ridge regression analysis with the CRFS as the outcome. The strongest contributors to hybrid index were vegetables, sweets/fat/refined carbohydrates, and complex carbohydrates. The vegetables contributed to a healthier CRFS, while both the sweets/fat/refined carbohydrates and complex carbohydrate groups contributed to a less healthy CRFS. These findings add to evidence that merely asking individuals what they like or dislike to eat can aid understanding of diet-disease relationships through capturing a usual pattern of dietary behaviors with potential dietary implications for young adults who show a risk for future metabolic syndrome.

Part of the utility of a diet quality index is how dietary intake is estimated. Dietary assessment methods aim to measure usual consumption, with minimal measurement error and bias [19]. Most diet quality indexes are derived from 24-h food recalls, which reflect dietary intake over the short-term, or food frequency surveys, which reflect dietary intakes usually over 1 year [36]. Each of these two methods can be time intensive, memory-demanding, cognitively challenging, and are subjective to measurement errors [19]. Indeed, a recent study found no association between reported diet quality intake derived from a 24-h food record (i.e., short-term intake) and measures of cardiometabolic risk in young adults [14]. Food liking surveys are easy for participants to complete in a short amount of time with low cognitive and memory burden [54,55], both of which encourage participation. However, hedonic ratings can show bias. For example, individuals with higher levels of dietary restraint report a lower liking for less healthy foods [56] and using both self-reported liking and intake can identify misreporting and improve intake of less liked foods [21,30,35]. Self-reported food likes and dislikes appears to capture habitual dietary intake. Food likes connect markers of taste genetics to intake [57,58] or health [30], correlate with self-reported intake [21,27,28,29,30,31], and associate with biomarkers of consumption [21,32,33] and health outcomes [20,21,22,23,24,32,33].

Another important consideration of diet quality indexes is the scoring and weighting of the individual components, particularly in the examining of associations with cardiovascular disease risk [15]. The Healthy Eating Index scoring is complex, with significant dietary analysis to derive food group scores representing the whole diet, determining portion equivalents adjusted for total energy intakes, and weighting according to the Dietary Guidelines as the relevant standard [18]. In comparison, the scoring of the theoretical, liking-based diet quality index is analytically simple—averaging within the food and beverage groups, weighting, and averaging into the index, while the hybrid, liking-based diet quality index requires a more sophisticated analysis to derive food and beverage group weights based on the associations with the cardiometabolic risk factor scores. Our previous research has shown that the liking-based diet quality index was not associated with energy intake [30,39]. Utility of diet quality indexes also have been graded on their validity and reliability [59]. The liking-based diet quality indexes in the present study and others [53,60,61] have good variability, approaching normal distribution, construct validity, and internal reliability. Combining liking-based with frequency-based diet quality indexes can increase the precision in predicting diet-disease relationships [30,39] through indirect identification of dietary restraint (intake is less than level of liking) and of behavior change (intake is greater than level of liking).

Criterion validity of the liking-based diet quality indexes in the present paper and others [53,60,61] is supported through ability to distinguish diet quality between patients with diagnosed depression versus controls as well as associations with the CRFS. We found lower diet quality across all of the diet quality indexes among those with depression, which agrees with previous literature (e.g., [62,63,64]). The results of this study are similar to those quantifying diet through traditional methods, such as a 24-h recall or food frequency questionnaire [65,66]. For example, among adolescents in NHANES, the prevalence of the metabolic syndrome decreased incrementally by each quartile of the Healthy Eating Index [67]. Similarly, in a sample of over 2000 Iranian men and women, adherence to the U.S. Dietary Guidelines significantly reduced the prevalence of the metabolic syndrome [66]. In the Framingham Heart Offspring Study, quintile of adherence to the Dietary Guidelines also reduced the incidence of metabolic syndrome in participants under the age of 55 years [68]. Each of the studies quantified dietary intake through intensive methods, including 24-h dietary recall (NHANES) or food frequency questionnaire.

The present study found that the hybrid derived diet quality index produced the strongest associations with the cardiometabolic risk factor score, with greatest weights for the liking for vegetables (liking associated with less cardiometabolic risk) and carbohydrate and fat foods (sweets, saturated fats, refined and complex carbohydrates associated with greater cardiometabolic risk). This finding agrees with previous studies of metabolic health and diet quality with improved associations by using a data-driven diet quality score over *a priori* [69] or analysis of population-based data to clarify recommendations through a hybrid approach of conceptually-based food groups and patterns identified through reduced rank regression analysis [70]. Hybrid methods benefit from *a priori* methods with the underlying structure of dietary patterns [37]. The hybrid method allowed validation of the weighting of the conceptual food/beverage groups as recommended [15] and reduced the importance of 10 weighted groups in the theoretically-constructed DQI to a smaller number of food groups. Our hybrid diet quality index was derived from ridge regression analysis, which has been supported to produce a greater association with cardiovascular health than analysis with principal component analysis [42]. Our findings are consistent with a dose-response meta-analysis of prospective studies that reports beneficial associations between vegetable intake and better cardiometabolic health [71]. In addition, our finding of an association between sweet, fat, and refined carbohydrates and greater CRFS agrees with sugar intake and a dose-response meta-analysis of prospective cohort studies [72]. We identified a common theme across sweet and refined carbohydrates and CRFS, which may not reveal a recent finding of a cardiovascular benefit of replacing sugars with starch in the diet [73]. However, total carbohydrate intake was associated with mortality from cardiovascular disease in a prospective study across multiple countries [74]. The association between fat and metabolic health is controversial and lowering fat among young adults may not improve metabolic health [47].

Metabolic syndrome is common even in young adulthood, doubling the risk for cardiovascular disease and increasing risk for diabetes five-fold [75]. Young adulthood further appears to be a key transition time for health consequences of metabolic risk, as a high metabolic risk score at the age of 25, or a worsening score from ages 25–40, predicted two–three times the likelihood of coronary artery calcification at year 25 [76]. In this study, we generated a metabolic risk score that was comprised of fasting insulin, glucose, HDL cholesterol, triglycerides, BMI, and waist-to-hip ratio to examine the relationships with DQIs. This particular clustering of metabolic risk factors deviates from previous trials who have found significant loading factors for blood pressure in addition to insulin, glucose, lipids, and BMI in adults [5,77,78,79]. Only 13% (*n* = 28) of our sample qualified as having hypertension, leaving open the possibility that there were too few cases of hypertension to detect important risk relationships with blood pressure or the relative importance of blood pressure on cardiometabolic risk in young adults is less than that of other factors [79,80,81,82]. We replicated findings that support insulin, glucose, and obesity as core determinants of cardiometabolic risk [3]. These factors consistently associate with development of CVD, type 2 diabetes, and morbidity and mortality rates across the lifespan [78]. Moreover, it is currently thought that the underlying cause of metabolic syndrome originates from dysregulation in insulin and glucose handling in response to adiposity, and therefore, are often the first risk factors to present clinically (followed by dyslipidemia and hypertension) [3,83,84,85]. Collectively, our findings suggest that young adults exhibit a ‘metabolic syndrome’ phenotype that likely represents only the core features of metabolic dysfunction and differs from that of later adulthood (i.e., inclusion of blood pressure) [4,85,86]. Additional studies that further investigate the variability in clustering of cardiometabolic risk factors from young adulthood to later adulthood are warranted [5].

It is estimated that nearly half (~47%) of all U.S. adults have at least one cardiometabolic risk factor, a statistic that differs in our sample [87]. Indeed, 65% (*n* = 138) of participants in the study had the presence of at least one metabolic abnormality. This discrepancy may be due to the disproportionate number of participants with major depressive disorder; a population known to have metabolic disturbances [88]. Fifty percent of the sample had diagnosed major depressive disorder. National data reports 10.9% of adults have diagnosed depression; however, the rates among college students are considerably higher (~40%) and more similar to this study of college-aged adults [89]. Our sample showed rates of dyslipidemia (14% versus 12%), hypertension (13.2% versus 18%), obesity (14% versus 36%), and pre-diabetes (4.2% versus 16.0%) at generally lower incidence than of the United States young adult population (20–39 years), with the exception of depression [80]. Our study population characteristics were similar to that of Williams et al., who also examined metabolic syndrome, diet quality and relationships with cardiometabolic risks in a sample of college-aged participants [14]. Despite these similarities, our findings did not replicate previous conclusions about diet quality and cardiometabolic risk [14]. The methodological approach applied in this study has been suggested as a more sensitive and specific mechanism to examine cardiometabolic risk relationships and contributed to our ability to uncover these important relationships [4,5,6]. Our findings are translatable to the general young adult population and provide important methodological tools that better assess chronic disease risk in young adults.

The present study has a number of limitations, which introduces future avenues of research. First, while the relative importance of the food groups from the most predictive DQI (i.e., hybrid DQI) had moderate consistency with the U.S. Dietary Guidelines, the statistically generated weights for some food groups had small/insignificant but opposite directions from these guidelines, such as alcohol, low-fat dairy, and healthy fat. This could be due to the instability of coefficients during the model training for less important food groups when using a relatively small sample size (~200). Future research may include a larger sample to further investigate the relationship between each food group and the cardiometabolic risk factor score. Second, while we established a significant association between the food-liking based DQI score and the cardiometabolic risks, caution should be exercised when generalizing our results to the general population. All of the study subjects in our sample were young adults, and only 13% of them were diagnosed with hypertension, which partly explains why blood pressure did not contribute to the principle cardiometabolic risk factor score (CFRS) in our sample. In addition, half of the study subjects had diagnosed depression, which was much higher than the prevalence of depression among US adults ages 18 to 25 years old (13.1%) [90]. Thus, the composition of CFRS and the relative importance of each component in DQI from this study may not be generalized to general population and future study with larger and more representative sample of US adults is warranted. Finally, while we standardized the hybrid DQI to allow for direct interpretation of the clinical importance of each food group, we could not directly assess its validity by comparing it with other established dietary quality measures (e.g., HEI and 24-h dietary recall) on the same subject. Future studies may better compare these measures by constructing dietary quality scores via different methods on the same group of subjects.

## 5. Conclusions

In this pilot study, we constructed several dietary quality indexes (DQI) from a novel food-liking survey and assessed their associations with a continuous cardiometabolic risk factor score (CRFS) based on plasma (insulin, glucose, lipids) and adiposity (BMI, Waist-to-Hip ratio) measures for a group of young adults. Among them, a hybrid DQI, comprising theoretically generated and internally reliable nutritional groups, as well as empirically generated weights, significantly predicted the CFRS in out-of-sample validations. Our results point to the protective effects of vegetable liking as well as the detrimental effects of sweet, saturated fat, and carbohydrate liking on cardiometabolic risks. Survey-reported liking and liking-based diet quality indexes can be feasibility added to research studies where full dietary assessment is not practical or as additional diet assessment measure. Future research is needed to further validate our study results using larger samples and continue to compare these novel measures with other established diet quality measures.

## Figures and Tables

**Table 1:** Food groups generated from a food liking survey, listed from most to least internally consistent assessed by the Cronbach’s alpha.

GROUP	FOOD GROUP ITEMS	Alpha
High-Fat Protein	Sausage, hotdog, beef steak, fried chicken, bologna, bacon	0.79
Refined Carbohydrate	Rice, bagels, pasta, cracker, pizza	0.742
Sweets/sugary beverages	Ice cream, cookies/cakes/pastries, cake icing, cheesecake, chocolate milk, soda, sweetened coffee drink	0.723
Healthy Fat/seafood	Tuna, salmon, baked fish, shrimp/other shellfish, olive oil	0.716
Fruit	Blueberry, melon, strawberry, mango, pineapple	0.713
Alcoholic beverages	Wine, scotch, dark beer	0.689
Vegetable	Broccoli, carrots, greens, sweet potato, mushrooms, tomatoes, tomato juice	0.687
Spicy/flavorful	Horseradish/wasabi, burn of a spicy meal, tabasco sauce, soy sauce, grapefruit juice, black coffee, dark chocolate	0.663
Saturated fat	Mayonnaise, whole milk, full fat dressing, cheddar cheese	0.654
Salty	Soup, lean ham, baked chicken, chips, salty pretzels, French fries	0.608
Complex carbohydrate	Whole wheat bread, oatmeal, shredded wheat cereal	0.509
Low-Fat Dairy	Low-fat cottage cheese, skim milk, plain yogurt	0.435

**Table 2:** Demographic and cardiometabolic risk factors in young adults who were patients with diagnosed depression or students at a university.

	Patients (*n* = 106)	Students (*n* = 106)	T-Value, Chi Squared, or Mann-Whitney U Z
**Gender**			χ^2^(1) = 0.321
male (*n* = 80)	39.6%	35.8%	
female (*n* = 132)	60.4%	64.2%	
**Age (years, mean ± SEM)**	21.5 ± 0.21	20.3 ± 0.13	Z = 4.029 **
**Body mass index (kg/m^2^) (mean ± SEM)**	27.4 ± 0.9	23.2 ± 0.7	Z = 3.388 **
underweight (<18.5) (*n* = 9)	4.7%	3.8%	χ^2^(1) = 8.732 **
normal (18.5–24.9) (*n* = 120)	46.2%	67%	
overweight (25–29.9) (*n* = 54)	23.6%	27.4%	
obese (>30) (*n* = 29)	25.5%	1.9%	
**Waist: Hip Ratio (mean ± Sem)**	0.79 ± 0.6	0.86 ± 0.7	Z = 7.60 ***
**Blood pressure (BP)**			
diastolic mmHG (mean ± SEM)	72.16 ± 1.07	71.20 ± 0.79	Z = 0.75
systolic mmHG (mean ± SEM)	112.81± 1.58	110.56 ± 1.21	Z = 0.98
**Total cholesterol mg/dL**	169.4 ± 3.47	166.3 ± 2.74	T = −0.563
LDL-C mg/dL †	93.3 ± 3.01	88.8 ± 2.41	T = −1.009
HDL-C mg/DL †	54.4 ± 1.49	61.2 ± 1.43	T = 3.425 **
cholesterol:HDL ratio †	3.33 ± 0.11	2.85 ± 0.07	T = −3.549 **
**Triglycerides mg/dL**	108.9 ± 5.93	82 ± 3.24	T = −3.783 **
**Fasting blood glucose mg/dL**	91.4 ± 0.87	87.2 ± 0.77	Z = 3.156 **
**Insulin mIU/L**	17.8 ± 2.27	9.6 ± 0.46	Z = 3.723 **

* *p* < 0.05; ** *p* < 0.01; *** *p* < 0.001. † LDL-C= low density lipoprotein cholesterol; HDL-C = high density lipoprotein cholesterol.

**Table 3:** Descriptive statistics and pairwise correlations among all nutritional groups.

Variable	Fruit	Refined Carbohydrate	Sweet	Salty	Healthy Fat	Saturated Fat	Vegetable	High-Fat Protein	Complex Carbohydrate	Spicy/Flavorful	Alcohol	Low-Fat Dairy
Mean	46.29	40.45	33.37	26.01	11.01	10.02	5.44	5.08	2.51	−5.59	−10.28	−11.54
Std. Dev.	33.13	29.02	30.09	28.31	40.89	39.58	34.19	41.36	36.23	34.75	51.63	39.20
Min	−96.5	−61.2	−94.25	−79.66	−96.8	−97.5	−96.16	−100	−100	−87.28	−100	−100
Max	100	100	100	100	91.4	100	91	93.33	100	72.85	97.66	100
Fruit	1	0.06	−0.06	−0.01	0.13	0.03	0.43 ***	−0.13	0.34 ***	0.21 **	0.18 *	0.32 ***
Refined Carbohydrate		1	0.67 ***	0.55 ***	−0.002	0.55 ***	0.003	0.28 ***	0.048	0.036	0.10	−0.004
Sweet			1	0.467 ***	0.041	0.590 ***	−0.144 *	0.342 ***	−0.065	0.067	0.097	0.006
Salty				1	0.245 ***	0.369 ***	−0.063	0.664 ***	−0.066	0.133	0.021	0.0015
Healthy Fat					1	0.133	0.276 ***	0.326 ***	0.164 *	0.309 ***	0.124	0.213 **
Saturated Fat						1	0.041	0.375 ***	0.0005	0.168 *	0.135	0.185 **
Vegetable							1	−0.148 *	0.447 ***	0.393 ***	0.125	0.335 ***
High-Fat Protein								1	−0.125	0.161 *	0.086	−0.057
Complex Carbohydrate									1	0.26 ***	0.165 *	0.417 ***
Spicy/Flavorful										1	0.305 ***	0.392 ***
Alcohol											1	0.223 **
Low-Fat Diary												1

* *p* < 0.05; ** *p* < 0.01; *** *p* < 0.001.

**Table 4:** Food groups generated from a liking score in young adults that formed the Hybrid Diet Quality Index (DQI) listed as those associating with better (adequacy) and worse (moderation) cardiometabolic risk factor score (CRFS). Food groups were weighted according to strength of the association with the CRFS to achieve a 0 to 100-point score.

Component	Maximum Points	Liking Score for Maximum Score	Liking Score for Minimum Score of Zero
Adequacy			
Vegetables	18	100	−100
Alcohol	3	100	−100
Fruit	2	100	−100
Moderation			
Sweet, Fat, and Refined Carbohydrates	52	−100	100
Complex Carbohydrates	15	−100	100
Spicy/Flavorful	4	−100	100
Low-Fat Diary	3	−100	100
Salty and High Fat Protein	2	−100	100
Healthy Fat	1	−100	100
